# Failure of Therapeutic Anticoagulation in COVID-19 Patients With Acute Ischemic Stroke. A Retrospective Multicenter Study

**DOI:** 10.3389/fneur.2022.834469

**Published:** 2022-03-04

**Authors:** Francesco Janes, Gian Luigi Gigli, Fedra Kuris, Mauro Morassi, Paolo Costa, Lorenzo Nesi, Roberta Giacomello, Federico Mazzacane, Eleonora Leuci, Anna Cavallini, Mariarosaria Valente

**Affiliations:** ^1^Clinical Neurology Unit, Santa Maria della Misericordia University Hospital, Udine, Italy; ^2^Department of Medicine, University of Udine, Udine, Italy; ^3^Neuroradiology Unit, Fondazione Poliambulanza, Brescia, Italy; ^4^Neurology Unit, Fondazione Poliambulanza, Brescia, Italy; ^5^Emergency Neurology Unit and Stroke Unit, Istituto di Ricovero e Cura a Carattere Scientifico (IRCCS) Fondazione Mondino, Pavia, Italy

**Keywords:** COVID-19, ischemic stroke, therapeutic anticoagulation, coagulopathy, large vessel disease

## Abstract

**Background:**

Acute ischemic stroke (AIS) is a possible complication of coronavirus disease 2019 (COVID-19) infection. Although peculiar clinical features and underlying specific mechanisms of thrombogenesis have been suggested so far, there is no consensus on the appropriate vascular preventive drug regimen in patients with COVID-19.

**Aim and Methods:**

From a larger clinical series of consecutive acute ischemic strokes related to COVID-19 admitted to three cerebrovascular units in Northern Italy, herein, we describe the clinical features of a subgroup of patients in whom stroke occurred despite therapeutic anticoagulation.

**Results:**

A total of seventeen/80 AIS related to COVID-19 (21.2%) occurred in anticoagulated patients. Although no blood level was available for Direct Oral AntiCoagulant, the drug dosage was appropriate according to guidelines. Their National Institute of Health Stroke Scale (NIHSS) at admission was 12.0 (SD = 7.4) and 58.8% of them had evidence of large vessel occlusion. The case fatality rate was as high as 64.7%.

**Discussion and Conclusions:**

The occurrence of an anticoagulation failure seems to be increased in the setting of COVID-19 infection, with worse clinical outcomes if compared to non-COVID-19 related ischemic strokes. We discuss the diagnostic and therapeutic implications of such evidence, suggesting that some arterial thrombotic complications might be either resistant to or independent of the anticoagulation effect.

## Introduction

Acute Ischemic Stroke (AIS) has been so far reported to complicate 1–5% of coronavirus disease 2019 (COVID-19) infections (1, 2). Specific clinical and radiological features were described in clinical series as well as in case-control studies of COVID-19-related strokes. They include a younger age of onset, worse clinical outcome, higher proportion of large vessel occlusion, frequent multifocal involvement in the COVID-19 group, and, to some extent, a relationship with COVID-19 severity itself (3–7). Although specific mechanisms of thrombogenesis (e.g., the inflammatory storm effect, a hyper-coagulation state, a diffuse endothelial activation/damage, and an impaired fibrinolysis state) have extensively been described (8, 9), guidelines on clinical management and already published papers do not provide evidence for a likewise specific preventive drug regimen in AIS related to COVID-19. At the same time, the pros and cons of anticoagulation in the prevention of both arterial and venous thrombotic complications in COVID-19 are still under debate (10).

In the general population, 5–10% of all AIS are known to occur despite anticoagulation in patients with non-valvular atrial fibrillation (NVAF) (11, 12). Few papers have analyzed the reasons for anticoagulation failure. A relevant proportion of those patients usually takes a subtherapeutic dose of anticoagulant, both, if Anti Vitamin K (AVK) and Direct Oral Anticoagulants (DOACs) are considered (13). However, the same authors suggest that, in patients with adequate compliance and dosage, other mechanisms for the development of AIS should be taken into consideration. In a study from South Korea (14), ischemic stroke recurrence was higher in “patients with NVAF with AIS on prior anticoagulation” than in those “ones without prior anticoagulation.” Authors discuss that only a part of this higher risk is explained by the non-compliance to anticoagulation treatment and that other causes might explain it (such as concurrent cardiomyopathy, malignancy, or atherosclerotic pathology). AISs on anticoagulation were found to be associated with a lower baseline National Institute of Health Stroke Scale (NIHSS) score (12) and with a better functional outcome at 3 months (15, 16).

The aim of this paper is to report the peculiar clinical features of several observed cases of AIS events that occurred in anticoagulated patients with COVID-19 during the last year of the pandemic and to discuss their clinical and therapeutic possible implications.

## Materials and Methods

### Patients' Selection Criteria

**Figure 1:**
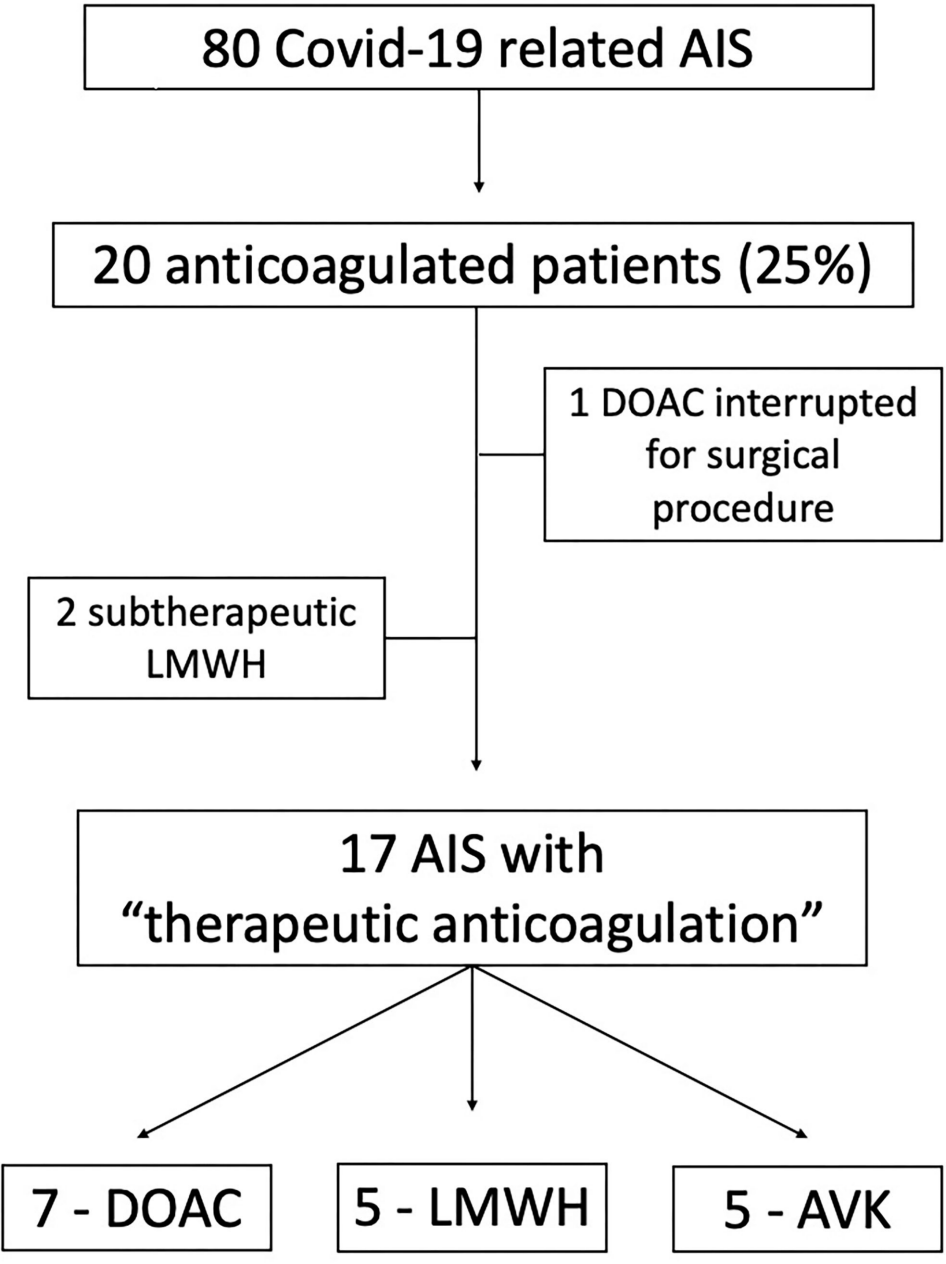
Flowchart diagram of the patients' selection process. AIS, Acute Ischemic Stroke; DOAC, Direct Oral Anticoagulant; LWMH, Low Molecular Weight Heparin; AVK, Anti K Vitamin anticoagulant.

We selected and pooled together the AISs occurred despite therapeutic anticoagulation among all the stroke events observed in patients with COVID-19 admitted and treated in three Neurology Units in Northern Italy, between March 2020 and April 2021. Patients with subtherapeutic and erroneous prescriptions were excluded from this series. AISs related to anticoagulation interruption—for instance, due to a diagnostic procedure or surgery—were excluded as well. In Figure 1 we show the patients' selection process.

Patients were considered eligible for inclusion in this series independently of the specific indication to anticoagulant therapy (e.g., for atrial fibrillation prevention or pulmonary embolism treatment).

The diagnosis of COVID-19 infection was confirmed in all patients through Reverse transcriptase viral PCR testing. Patients whose test became positive >72 h after admission or stroke onset were excluded from this series.

### Therapeutic Anticoagulation Definition

“Therapeutic anticoagulation” was defined as an International Normalized Ratio (INR) > 2.0 in the case of AVK drugs. For patients in DOAC, it was defined if the dosage was appropriate following the prescription guidelines (age, body weight, GFR, concurrent drugs…) or, when available, if the plasma levels fell within therapeutic range at the time of stroke. In the case of patients on low molecular weight heparin LMWH, the therapeutic dose was defined as the starting dose of 1 unit/kg (17).

### Variables and Risk Factors Definition

High blood pressure (HBP) was defined as the systolic pressure of 140 mmHg, diastolic pressure of 90 mmHg, use of antihypertensive medication, and/or being told at least twice by a physician or other health professional that one has HBP. Atrial fibrillation (AF) was diagnosed if the patient had an AF in the ECG prior to stroke or in the continuous Electrocardiogram (EKG) monitoring recording during hospitalization. The Trial of ORG 10172 in Acute Stroke Treatment (TOAST) criteria were used to classify stroke etiology, into the following 5 groups: Large Artery Disease (LAD); Cardioembolic (CE); Small Vessel Disease (SVD); undetermined (UND); Other determined etiology (OTH) (18). Stroke due to a large vessel occlusion (LVO) was defined if there was evidence of occlusion/severe stenosis of an extracranial epi-aortic artery, intracranial internal carotid artery, middle cerebral artery (both M1 and M2 tracts); evidence of aortic atheroma > 1.5 mm was considered qualifying for a large vessel stenosis/occlusion and LAD in the TOAST category.

The NIHSS and the modified Rankin Scale (mRS) were used as markers of stroke severity and outcome.

COVID severity was classified according to the WHO severity index (0 = asymptomatic; 1 = mild with no pneumonia; 2 = moderate pneumonia; 3 = ARDS and critically ill) (19).

### Statistical and Ethical Issues

The available clinical features are summarized for each patient and the descriptive statistics for the overall series are reported as mean ± SD for continuous variables, after checking for normality of their distribution with the Shapiro-wilk test. Categorical variables are described as the proportion (%) out of the total. Statistical analysis was performed with SPSS 27.0 (IBM Corporation; New Orchard Road Armonk, NY 10504).

The study was promoted by the Clinical Neurology Unit of the Azienda Sanitaria Universitaria Friuli Centrale (ASUFC) in Udine and approved by the regional E.C. and local ones at collaborating sites (approval# CEUR-2021-os-117). The patients' data were managed according to the Helsinki declaration and good clinical practice principles.

## Results

Altogether, the three Units admitted 80 COVID-19-related AISs during the specified period (40 in Udine, 21 in Pavia, and 19 in Brescia). A total of seventeen patients/80 (21.2%) fulfilled the selection criteria; 16 with an ischemic stroke and 1 patient with a transient ischemic attack (TIA).

Their characteristics are summarized in Table 1. Noteworthy, most of them were male (76.5%) and 13/17 (76.5%) were functionally independent before stroke as measured by mRS. The baseline NIHSS was 12.0 (SD 7.4), 58.8% had an LVO and 41.2% were classified after diagnostic work-up as LAD strokes. Among patients, 35.3% had a severe form of COVID pneumonia requiring intensive care unit (ICU) management, 52.9% a moderate one, and 11.8% a mild to asymptomatic form.

**Table 1:** Clinical characteristics, risk factors, and outcomes of COVID19-related acute ischemic strokes (AISs) under therapeutic anticoagulation.

**ID**	**Age**	**Sex**	**mRS pre-**	**NIHSS at onset**	**Stroke subtype**	**Stroke location/territory**	**LVO**	**TOAST**	**Reperf**	**1 m/mRS**	**3 m/mRS**	**COVIDseverity**	**AF**	**HTN**	**Diab**	**Smoke**	**HyperCole**	**CHF**	**Indic for anticoag**	**Type of anticoagulant**	**Anticoag management**	**INR**	**PLT**
BS-01	72	F	0	0	TIA	–	No	UND	No	0	0	2	Known	Yes	No	No	No	No	CAF	VKA (warfarin)	Kept up	2,.52	115
BS-02	68	M	2	8	FEIS	r-MCA-M3; r-cerebellar	No	CE	No	6	6	3	Known	Yes	No	Former	Yes	Yes	PAF	VKA (warfarin)	Susp	2.8	46
BS-03	84	F	4	21	FEIS	l-MCA-M1	Yes	LAD	No	5	5	3	Known	Yes	No	No	Yes	Yes	CAF	DOAC (dabigatran 110 bid)	Susp	1.19	211
BS-04	72	M	2	16	FEIS	Cerebellar	No	UND	No	5	5	2	Known	No	No	No	Yes	No	PAF	VKA (warfarin)	Susp	2.21	240
BS-05	83	M	2	14	FEIS	r-MCA-M2; r-cerebellar	No	CE	No	6	6	3	Known	Yes	Yes	Current	Yes	Yes	PAF	VKA (warfarin)	Susp	4.03	212
BS-06	80	M	0	25	FEIS	r-MCA-M1	Yes	LAD	No	6	6	2	Known	Yes	Yes	No	No	No	CAF	VKA (warfarin)	Susp	4.05	218
PV-01	78	M	3	25	FEIS	l-MCA-M1	Yes	LAD	No	6	6	3	Known	Yes	No	Former	Yes	No	PAF	DOAC (dabigatran 110 bid)	-	0.7	–
PV-02	70	M	0	4	RIS	r-MCA-M1	Yes	LAD	IVT	3	2	2	Known	Yes	Yes	Former	Yes	No	PAF	DOAC (edoxaban 60)	Switch to other	–	–
UD-01	86	M	2	6	RIS^*^	l-PCA-P1	Yes	CE	No	6	6	3	new/o	Yes	No	No	No	No	PE	DOAC (rivaroxaban 15)	Kept up	1.18	162
UD-02	87	F	2	12	FEIS	Deep lacune	No	UND	No	6	6	0	Known	Yes	Yes	No	Yes	No	CAF	DOAC (edoxaban 30)	Susp	1.13	502
UD-03	76	M	3	13	FEIS	Deep lacune	No	SVD	No	5	4	1	No	No	Yes	No	No	No	PE	DOAC (apixaban 5 bid)	Kept up	1.45	164
UD-04	79	M	0	7	FEIS	Multi focal	Yes	LAD	No	6	6	2	No	Yes	Yes	No	No	No	PE	LMWH (enoxaparin 6,000 IU bid)	Switch to other	1.47	201
UD-05	71	M	0	19	FEIS	r-ICA	Yes	LAD	No	6	6	3	Known	Yes	Yes	Former	Yes	No	CAF	DOAC (dabigatran 150 bid)	Susp	1.46	294
UD-06	62	M	0	4	FEIS	r-MCA-M2	Yes	UND	No	6	6	2	No	No	No	No	No	No	PE	LMWH (enoxaparin 8,000 IU bid)	Kept up	1.22	220
UD-07	62	M	0	6	FEIS	Anterior circ	–	UND	No	4	4	2	No	Yes	Yes	No	Yes	No	PE	LMWH (enoxaparin 8,000 IU bid)	Kept up	0.97	385
UD-08	56	F	3	9	FEIS	l-MCA	Yes	OTH	No	6	6	2	No	Yes	No	No	No	No	PE	LMWH (enoxaparin 10,000 IU bid)	Kept up	1.06	164
UD-09	66	M	0	3	FEIS	l-ICA	Yes	LAD	No	6	6	2	No	Yes	Yes	Former	Yes	Yes	AMI	LMWH (enoxaparin 7,000 IU bid)	Kept up	1.19	107
Overall *n* (%) or mean ± sd	73.6 ± 9.1	F 23.5%	1.3 ± 1.4	12.0 ± 7.4	FEIS 82.3% RIS 11.8% TIA 5.9%	–	LVO 58.8%	LAD− 41.2% CE− 23.5% SVD− 5.9% OTH− 5.9% UND− 29.4%	IVT 5.9% MT 0,.0%	Death 64.7% Alive only 3.7 ± 2.0	Death 64.7% Alive only 3.3 ± 2.0	2.2 ± 0.8	Known 58.8% new/o 5.9% No 35.3%	82.4%	52.9%	Current 5.9% No 64.7% Former 29.4%	64.3%	23.5%	PAF− 29.4% CAF− 29.4% PE− 35.3% AMI−5.9%	AVK− 29.4% DOAC− 41.2% LMWH− 29.4%	Kept up− 43.8% Susp− 43.8% Switch to other− 12.4%	–	216.1 ± 112.3

*mRS, modified Rankin Scale; FEIS, first ever ischemic stroke; RIS, recurrent ischemic stroke*;

* *second IS during hospitalization; TIA, transient ischemic attack; MCA, middle cerebral artery; ACA, anterior cerebral artery; PCA, posterior cerebral artery; ICA, internal carotid artery; LVO, Large Vessel Occlusion; CE, cardioembolic; LAD, Large Artery Disease; SVD, small vessel disease; UND, undetermined; OTH, other etiologies; IVT, intravenous therapy; MT, mechanical therapy; CHF, Chronic Heart Failure; PE, pulmonary embolism; PAF, paroxysmal atrial fibrillation; CAF, chronic atrial fibrillation; AMI, acute myocardial infarction; BMI, Body Mass Index; VKA, Vitamin-K-Antagonists; DOAC, Direct Oral Anticoagulants; LMWH, Low Molecular Weight Heparin; PLT, Platelets count; Cr, creatinine*.

A total of seven patients (41.2%) had an already known COVID-19-pneumonia, 5 of them hospitalized (in detail: 2 for COVID-19-pneumonia and concurrent Pulmonary Embolism (PE), 1 for COVID-19-pneumonia plus first-ever stroke, 2 for COVID-19-pneumonia and they were taking DAOC for previous PE) and 2 were treated at home. The other 10 patients (58.8%) were hospitalized for AIS and were found positive for COVID-19 infection.

A total of seven out of 17 patients were treated with DOAC. None of them had plasmatic levels of DOAC available. Five out of 17 patients took AVK (Warfarin in all of them) and their mean INR at stroke onset was 3.12 (SD = 0.86); no AVK patients had an INR below 2.0. Five out of 17 patients were treated with LMWH (enoxaparin in all patients). Unfortunately, none of them had an anti-factor Xa level available at the time of stroke onset; 3 of them have a normal aPTT (activated partial thromboplastin time; reference range: 0,80–1,20) within 24 h from stroke onset, 1 a prolonged aPTT (1.74), and one more patient had no laboratory monitoring performed.

The mortality rate at 1 month after stroke onset was very high at 64.7%; In 8/11 patients (72.7%), the cause of death was a respiratory failure in moderate to severe pneumonia; in one patient, death was caused by the consequences of the severe stroke in the basilar artery territory; in two more patients, the cause was due to a multiorgan failure. Among the 5 survivors, only 1 patient had an mRS ≤ 2 at 3 months.

## Discussion

In this clinical series, we basically found that AISs occurring during COVID-19 infection despite therapeutical anticoagulation have clinical features different from those we already know from the literature. In fact, they show high baseline NIHSS, bad outcomes, and a very high mortality rate; the rate of large vessel disease is also very high.

To our knowledge, only one case report by Shoukry and Kite in the UK (20) already described a similar clinical scenario. Their case was an 89 male on Rivaroxaban with a moderate COVID-19 infection, in which AIS was due to an extensive LAD, leading to death within a few days. Noteworthy, this patient shared the same overall features of our series.

The proportion itself of anticoagulation failure seems to be higher in patients with COVID-19 if compared with published data, although it is not easy to estimate from the literature. Among the 5–10% of AISs occurring on anticoagulation, 2/3 are associated with drug prescription/management errors, often characterized by a subtherapeutic dose (11, 13). In the report from the Swiss Stroke Registry (15), 41% of patients on AVK had an INR <1.7. These data lead us to believe that what we could call “true failure” (i.e., during anticoagulation at therapeutic dosages) seems to be responsible for nearly 1.5–5.0 % of AISs. In our pooled series of COVID-19-related AISs, the overall proportion of events on therapeutic anticoagulation was 21.2%. During the same period of this study, at the Udine University Hospital, the proportion of AISs in non-COVID patients related to therapeutic anticoagulation (the same features of the clinical series herein described) was 5.3%. This data is consistent with the above estimates, and it is almost four times lower than the proportion found in patients with COVID-19.

In previous studies, the proportion of LVO ranged from 39 to 44% of AISs on anticoagulation (13, 15). In our cohort, this proportion was as high as 58.8%.

Thromboembolism prophylaxis has been used from the beginning of the COVID-19 pandemic, but it became rapidly clear that routine prophylactic dose—usually of an LMWH—was often insufficient to prevent venous thrombosis in those patients (21).

Anticoagulation therapy was then—and is nowadays—widely used in the COVID-19 infection management, despite several areas of uncertainty regarding its benefit/harm ratio being unsolved (10). Lachant et al. reported that therapeutic anticoagulation was associated with a lower rate of thromboembolic complications (22). However, they do not specify what type of thrombotic complication they actively searched for, and a cerebral CT scan was performed only in a minority of patients. It is difficult, therefore, to draw conclusions on the effect of anticoagulation on the prevention of arterial thromboembolism. In a precocious Dutch report by Klok et al., 3 cases of ischemic stroke were reported during anticoagulation ranging from prophylactic dose to therapeutic dose (23), but it is not mentioned if those specific patients were currently on a therapeutic anticoagulation regimen. On the other hand, Kats et al. warned on the wide use of anticoagulation after they saw, precisely in AIS, a higher proportion (31 vs. 4%) of associated hemorrhagic stroke in severe cases of COVID-19 in comparison to mild cases (5). Within the currently accepted concept that patients with COVID-19 had a hyper-coagulopathy and an accelerated thrombosis, our patients' characteristics suggest, from the clinical point of view, that the mechanisms sustaining that process could be either resistant to or independent of anticoagulation itself. The hypothesis of a different pathogenetic mechanism is furtherly supported by the observation that 58.8% of patients were appropriately receiving anticoagulation treatment because of atrial fibrillation. Unfortunately, this conclusion cannot be demonstrated. The main limitation of our series, however, is the lack of systematic monitoring of anticoagulation effectiveness. In fact, we can be reliably self-confident that anticoagulation was “effective” only in the ones on AVK, as demonstrated by INR values. We cannot have the same certainty in judging patients on DOACs, due to the lack of plasma levels, and even more in patients on therapeutic LMWH, not monitored systematically with anti-Xa.

During the last year, several studies have analyzed and reviewed in deep the biochemical steps that bring to a venous and/or arterial thrombotic event in COVID-19 and the complex interplay between the inflammatory storm, coagulation cascade, endothelial activation/damage, platelet aggregation, and impaired fibrinolysis (8, 9). Mounting evidence is emerging that coagulation cascade activation follows directly the hyperinflammatory response (24) and that the role of endothelial damage and platelet activation is at least as important as the one of the coagulation cascade itself. Thus, anticoagulation might not be the appropriate therapy for all “hypercoagulative states,” while adjunctive antithrombotic therapies (e.g., synthetic serine protease inhibitors such as nafamostat mesylate and camostat mesylate; antithrombin; plasma exchange; Acetyl Salicylic Acid) seem to be necessary and even desirable in some patients (8).

Given the intrinsic limitations of a small size clinical series, we have no presumption of drawing conclusions on the mechanisms that could underline a hypothetical resistance to anticoagulation. However, we can suggest that “anticoagulation failure” in AIS during COVID-19 infection is not infrequent. Further studies should identify biological markers in patients at risk for arterial thrombotic events—such as stroke—to identify at-risk patients, for whom a more tailored antithrombotic therapy would be suitable.

Finally, arterial thrombotic events have been considered so far uncommon in other infection-associated coagulopathies (8). However, no other infectious disease was so extensively investigated as COVID-19. It is consequently possible that the lessons learned with the extensive biological studies carried out in COVID-19 will lead to reconsidering stroke pathogenesis in the case of other ischemic strokes associated with an anticoagulation failure. This is important to establish stroke secondary prevention, even more so since the evidence was published of an increasing rate of anticoagulation in AIS in the last decades (25).

## Conclusions

The proportion of AISs related to COVID-19 infection presenting as anticoagulation failure seems to be increased and their clinical features are peculiar if compared with published data in the general population. This suggests that some thrombo-embolic events—above all arterial thromboembolic events—in COVID-19 might be either resistant to or independent of the anticoagulation effect. Consequently, these data might promote a debate about adjunctive/alternative vascular preventive therapy in patients with COVID-19. Finally, in the general field of stroke care, the lights shed by ischemic strokes triggered by COVID-19 infection should stimulate studies on anticoagulation failure mechanisms to ameliorate drugs therapy regimens.

## Data Availability Statement

The raw data supporting the conclusions of this article will be made available by the authors, without undue reservation.

## Ethics Statement

The studies involving human participants were reviewed and approved by CEUR-FVG, Comitato Etico Unico Regionale–Friuli Venezia Giulia, at ARCS, Agenzia Regionale di Coordinamento Salute–Via Pozzuolo 330, 33100 Udine (IT). Written informed consent for participation was not required for this study in accordance with the national legislation and the institutional requirements.

## Author Contributions

FJ and GG: conceptualization. FJ and FK: methodology and formal analysis. FJ, MV, and GG: validation. RG, FK, LN, EL, FM, PC, and MM: investigation. FK, MM, and FM: data curation. FJ: writing—original draft preparation. FJ and GG: writing—review and editing. AC, MV, and GG: supervision. All authors have read and agreed to the published version of the manuscript.

## Conflict of Interest

The authors declare that the research was conducted in the absence of any commercial or financial relationships that could be construed as a potential conflict of interest.

## Publisher's Note

All claims expressed in this article are solely those of the authors and do not necessarily represent those of their affiliated organizations, or those of the publisher, the editors and the reviewers. Any product that may be evaluated in this article, or claim that may be made by its manufacturer, is not guaranteed or endorsed by the publisher.
